# The Value of Individual Screen Response Time in Predicting Student Test Performance: Evidence from TIMSS 2019 Problem Solving and Inquiry Tasks

**DOI:** 10.3390/jintelligence13070082

**Published:** 2025-07-06

**Authors:** Bin Tan, Okan Bulut

**Affiliations:** 1 Measurement, Evaluation, and Data Science, Faculty of Education, University of Alberta, Edmonton, AB T6G 2G5, Canada; 2 Centre for Research in Applied Measurement and Evaluation, Faculty of Education, University of Alberta, Edmonton, AB T6G 2G5, Canada; bulut@ualberta.ca

**Keywords:** response time, process data, profile analysis, within-person variability, individual differences

## Abstract

The time students spend on answering a test item (i.e., response time) and its relationship to performance can vary significantly from one item to another. Thus, using total or average response time across all items to predict overall test performance may lead to a loss of information, particularly with respect to within-person variability, which refers to fluctuations in a student’s standardized response times across different items. This study aims to demonstrate the predictive and explanatory value of including within-person variability in predicting and explaining students’ test scores. The data came from 13,829 fourth-grade students who completed the mathematics portion of Problem Solving and Inquiry (PSI) tasks in the 2019 Trends in International Mathematics and Science Study (TIMSS). In this assessment, students navigated through a sequence of interactive screens, each containing one or more related items, while response time was recorded at the screen level. This study used a profile analysis approach to show that students’ standardized response times—used as a practical approximation of item-level timing—varied substantially across screens, indicating within-person variability. We further decompose the predictive power of response time for overall test performance into pattern effect (the predictive power of within-person variability in response time) and level effect (the predictive power of the average response time). Results show that the pattern effect significantly outweighed the level effect, indicating that most of the predictive power of response time comes from within-person variability. Additionally, each screen response time had unique predictive power for performance, with the relationship varying in strength and direction. This finding suggests that fine-grained response time data can provide more information to infer the response processes of students in the test. Cross-validation and analyses across different achievement groups confirmed the consistency of results regarding the predictive and explanatory value of within-person variability. These findings offer implications for the design and administration of future educational assessments, highlighting the potential benefits of collecting and analyzing more fine-grained response time data as a predictor of test performance.

## 1. Introduction

The increasing use of computer-based assessments enables the automatic collection of not only answer accuracy but also rich process data, defined as any data automatically collected about students’ response process (Anghel et al. 2024). Process data include response times (the latency between item presentation and response), keystroke dynamics, clickstream sequences, and navigation patterns. Such process data has emerged as a critical source of insight into educational assessment, allowing researchers and educators to explore and understand not only what students know, but also how they engage in the test process and its relationship with their performance. For instance, response time data has been used to detect aberrant behaviors such as cheating (Man et al. 2018), disengagement or quitting (Ulitzsch et al. 2020a, 2020b), rapid guessing (Bulut et al. 2023b; Wise 2019), and low test-taking effort (Wise and Gao 2017), while also capturing persistence and problem-solving strategies (Yang and Ogata 2023; Liu et al. 2025).

While many studies (e.g., Castro et al. 2019; Goldhammer et al. 2014; Ratcliff et al. 2015) have explored the relationship between response time and performance, they have primarily focused on the correlation between response time and response accuracy at the item level. However, such item-level analyses may fall short of predicting overall test performance or providing a comprehensive understanding of students’ test-taking behaviors and cognitive processes. This limitation stems from the fact that the relationship between response time and accuracy can vary throughout the test. Such variation may reflect student-related factors such as shifts in time allocation strategies, motivation, test-taking behaviors, achievement levels, and cognitive resources over the course of the testing event (Bolsinova et al. 2017; Domingue et al. 2022; Schnipke 1995; Van Der Linden 2009; Wise and Kingsbury 2016), as well as task-related factors such as item difficulty, type, and position (Tan 2024; Van Der Linden 2009). Additionally, Wise and Ma (2012) found a negative correlation between response time and accuracy for easier items, but a positive correlation for more difficult ones. Goldhammer et al. (2014) showed that longer response times improved performance on problem-solving tasks, while shorter times were more beneficial for reading tasks. Differences in the response time–performance relationship have also been observed across cognitive domains (Chen et al. 2018; Krämer et al. 2023).

To predict overall test performance, some researchers rely on total response time or the average response time across items as predictors (e.g., Sherbino et al. 2012). However, this approach assumes that a student’s response time, relative to others, remains consistent across all items during the test, with no within-person variability. In other words, it presumes that if response time data are standardized, a student’s standardized response time does not fluctuate across items—for instance, they consistently spend more time than others on all items. However, this assumption is often untested and likely unrealistic. Ignoring the within-person variability in response time also results in the loss of information needed to predict overall test performance.

This study aims to address the limitations of the aforementioned practices for using response time to predict and explain overall test performance. We define within-person variability in response time as the fluctuation in a student’s standardized response times across test items or screens in a computer-based assessment. Instead of collapsing these fluctuations into a single summary statistic (e.g., standard deviation), we adopt a profile analysis approach that treats each student’s standardized response times at the item or screen level as a multivariate predictor profile. Specifically, the study has three main objectives: (1) to identify meaningful within-person variability in response time across items or screens, (2) to evaluate the predictive value of this variability for overall test scores above and beyond average response time, and (3) to explore the explanatory value of response time profiles by identifying patterns associated with higher performance. We argue that each item- or screen-level response time may relate to overall test scores in unique ways. Taken together, this study aims to demonstrate the importance of considering individual item-level response times and their variability in the collection and analysis of response time data in educational assessments.

## 2. Literature Review

This section is organized as follows. Section 2.1 reviews prior research on the relationship between response time and test performance, highlighting its complex, idiosyncratic, and context-dependent nature, which is influenced by many factors and reflects students’ test-taking process. Using students’ test-taking behaviors and cognitive processes as examples, we discuss how fluctuations in response times across items are likely to occur, and how ignoring such changes can lead to a loss of valuable information about students’ test-taking process. This underscores the importance of identifying within-person variability in response time. Section 2.2 turns to related methodological work, reviewing diverse existing approaches that have been developed and used to examine within-person variability in response time. We highlight that while these studies offer valuable insights, few have directly quantified the predictive value of within-person variability itself—a gap that this study aims to address. Therefore, in Section 2.3, we restate the study’s objectives and introduce profile analysis as a well-suited methodological approach to achieve them. Finally, we conclude the section by presenting the three research questions that guide our investigation.

### 2.1. The Idiosyncratic Relationship Between Response Time and Performance

Many studies have examined the relationship between response time and performance. However, they have reported inconsistent findings. Some report that faster responses correlate with better performance (e.g., Castro et al. 2019; Sherbino et al. 2012), while others find no clear relationship (Ratcliff et al. 2015), a positive relationship (Goldhammer et al. 2014), or a curvilinear one (Chen et al. 2018).

Further investigations revealed that many factors contribute to diverse response time patterns and moderate and explain the relationship between response time and performance (e.g., Wise 2006). For example, rapid guessing, in which students respond immediately without fully engaging (Schnipke 1995), can reduce the likelihood of correct answers, thus inflating the positive link between response time and performance (Wise 2006). Because students may switch between solution behavior (i.e., when a student strives to answer an item correctly by reading and considering it carefully) and rapid guessing in a test event (Wise and Smith 2011), the relationship between response time and accuracy can shift from item to item.

When students do engage in solution behaviors, changes in response time and their relationship with performance can be explained by Shiffrin and Schneider’s (1977) dual processing theory, which posits two cognitive processes involved in test completion: controlled processes and automatic processes. Controlled processes require cognitive resources and effortful mental operations, allowing students to respond to items and potentially arrive at the correct solution at a slower pace (Naumann and Goldhammer 2017). In this case, there is a negative relationship between response time and test performance. On the contrary, automatic processes are executed effortlessly and quickly and are unaffected by cognitive load, which usually reflects familiarity or excellence in performing the task. Thus, automatic processes imply a positive relationship between response time and test performance. Additionally, researchers found that faster responses correlate with better performance for easier items (Wise and Ma 2012). As item difficulty increases, more capable students employ more automatic processes than less capable students, resulting in faster response times (Sweller et al. 1998). Although students relying on controlled processes may take more time to arrive at correct solutions (Krämer et al. 2023), they may gradually struggle with more challenging items due to increased cognitive load and limited cognitive resources (e.g., fatigue). The exhaustion of cognitive resources may even result in unmotivated test-taking behaviors such as rapid guessing (Wise 2015), leading to fast response times but low performance (i.e., positive correlation).

As students’ test-taking behaviors and cognitive processes vary based on their abilities and the difficulty of test items throughout an assessment, their standardized response times are likely to fluctuate, reflecting within-person variability in response time. The idiosyncratic relationship between response time and performance also suggests that aggregate measures, such as total or average response time, may fail to accurately predict and adequately explain overall test performance, as they lose information about the relationship between response time and performance on individual items. Therefore, it is reasonable to hypothesize that considering within-person variability in response time across individual items could offer a more accurate prediction and a more informative explanation of overall test performance.

### 2.2. Related Work of Studying Within-Person Variability in Response Time

Several statistical approaches, such as hierarchical models (Van der Linden 2007), have been proposed and used to model the relationship between response time and performance. These models often assume no systematic within-person variation in response time or ability across items, implying that the relationship between response time and performance can be fully explained by speed (quantified by response time) and ability alone (i.e., conditional independence). In practice, however, these assumptions can be too restrictive and unrealistic, as shown in Section 2.1. For example, Bolsinova et al. (2017) found that although the overall correlation between speed and ability was near zero, “residuals” in response time—defined as deviations from expected values based on individual speed and item time intensity—systematically influenced the accuracy of responses. These residual patterns suggested that students adjusted their speed or employed different strategies across items, thus violating the conditional independence assumption.

Recently, a few studies have explored within-person variability in response time to better understand the relationship between response time and performance. Domingue et al. (2022) explored within-person variability in response time (indicated by changes in rank for time spent across items compared to peers) to determine whether additional time spent on an item yielded marginal gains in accuracy beyond the predicted probability of accuracy. They observed curvilinear and inconsistent relationships between response time and accuracy across 29 datasets. Moreover, they found that individuals with higher within-person variation in response time often exhibited lower ability, suggesting that added time does not necessarily translate into improved performance. Factors such as motivation and task difficulty significantly influenced these relationships.

Mutak et al. (2024) introduced the intraindividual speed–ability relationship (ISAR) model, which extends Van der Linden’s (2007) hierarchical framework by allowing both speed (quantified by response time) and ability to vary during a test. Their findings demonstrated that the direction and strength of the speed–ability relationship varied across individuals. While some exhibited a negative trade-off—faster speed coupled with lower ability—others displayed positive or curvilinear patterns.

More recently, Alfers et al. (2024) investigated within-person variability in response time and its link to accuracy through a within-subject experimental design. Using an extension of Van der Linden’s (2007) hierarchical models, they modeled latent speed and ability separately for conditions emphasizing response accuracy and for those emphasizing both speed and response accuracy. They then derived latent change scores to capture how participants shifted their performance strategies under changing priorities. This method enabled them to isolate individuals’ trade-offs between speed and accuracy and uncover meaningful within-person variations.

Unlike traditional models that treat response time as a static measure, Chen et al. (2019) introduced an event history analysis approach that models test-taking as a continuous-time process, allowing for real-time predictions of task duration and final success probability. By leveraging survival analysis techniques, they estimated the probability of success at any point in the test based on a student’s sequence of prior actions, rather than relying solely on aggregated response times. Their framework captures how response time patterns evolve dynamically, offering a more flexible and individualized perspective on test-taking behavior. This approach contributes to the field by providing a time-sensitive method to analyze response processes, revealing how within-person variability influences test performance over time rather than treating response time as a single, summary measure.

Tang et al. (2020) propose a novel approach to analyze within-person variability, focusing on process data as a whole rather than response time alone. Using multidimensional scaling, they extract latent features from high-dimensional action sequences, capturing behavioral patterns such as attentiveness and specific action intensities. Their method introduces a dissimilarity measure that quantifies differences between response processes based on the order and frequency of actions, without requiring prior knowledge of the actions’ meanings. This approach is computationally efficient and generic, applicable to diverse process data (including response time) without item-specific adjustments. Tang et al. demonstrate that the extracted features contain richer information than traditional binary responses, particularly for predicting performance such as numeracy and literacy scores.

The literature reviewed above contributes to the growing body of research demonstrating the existence of within-person variability in response time and its inconsistent relationship with both item-level and test-level performance. While prior studies have examined factors influencing within-person variability (e.g., ability, task difficulty, motivation, and performance strategies), the potential value of within-person variability itself remains underexplored. Specifically, few studies have explicitly quantified within-person variability in response time or investigated the extent to which considering this variability provides advantages over using total or average response time in predicting and explaining overall test performance.

### 2.3. Current Study: A Profile Analysis Approach

In earlier sections, we discussed the complexities in the relationship between response time and test performance and the research gap in the literature. To address these issues, this study adopts a profile analysis approach to identify within-person variability in students’ response time profiles and explores how this variability can predict and explain overall test performance.

Profile analysis refers to a set of multivariate data analysis techniques that focus on the shapes and patterns of profiles (Stanton and Reynolds 2000). Traditionally, profiles are viewed as vectors containing an individual’s or group’s subscores in an assessment, for example, subdomain scores in mathematics (e.g., algebra, geometry, arithmetic). By examining students’ performance across these subdomains, researchers can determine whether students perform consistently in each area, thus revealing their relative strengths and weaknesses (Bulut et al. 2017). As an illustration, a one-sample profile analysis with Hotelling’s *T*^2^ can be conceptualized as analyzing repeated measures to test within-subject equality of means, indicating whether scores remain constant across subdomains (Bulut and Desjardins 2020). Additionally, criterion-related pattern profile analysis has been used to examine the relationship between subscores and a specific external criterion, quantifying the proportion of variability in the criterion that can be explained by the level or pattern effect (Davison and Davenport 2002; Davison et al. 2015). The level effect corresponds to the predictive value of the average subscores, while the pattern effect considers the variability of the subscores within each profile. An example of the applications of criterion-related profile analysis is provided by Biancarosa et al. (2019), who show that students’ subscores in one achievement test add more predictive value than a single total score in predicting students’ scores on another achievement test.

In this study, we extend the logic of profile analysis by treating students’ screen-level response times during a test event as a profile. To achieve the study’s goals of identifying within-person variability in response time and examining its predictive and explanatory value for test performance, we address the following three research questions (RQs).

RQ1. Is there meaningful within-person variability in students’ response times across test screens?

This question examines the variability in students’ standardized response times across screens throughout a test event (i.e., within-person variability). Identifying this within-person variability lays the groundwork for determining whether separate screen-level response times are more predictive than the total or average response time across all screens. It supports the notion that students engage with different parts of the test in distinct ways, laying the foundation for the potential explanatory value of analyzing separate response times over total or average response time.

RQ2. To what extent does within-person variability in response time predict test performance beyond average response time, and does the relationship between response time and test performance vary across screens?

Building on RQ1, the second objective of this study is to assess whether within-person variability in response times (i.e., the pattern effect) adds predictive value beyond that of average response time (i.e., the level effect). Specifically, we decompose the total predictive value of response times into pattern and level effects and quantify the extent to which within-person variability contributes to the prediction of test performance. In addition, we identify how separate response times relate to test performance by identifying profile patterns associated with higher scores on test performance, referred to as criterion-related patterns. These criterion-related patterns allow us to explore how variability in response time across screens or items may reflect students’ test-taking behaviors and cognitive processes, all of which inform test-taking processes beyond what can be captured by total or average response time alone.

RQ3. Does within-person variability in response time consistently predict test performance more strongly than average response time across different achievement levels? How do individual screen-level response times relate to performance within each group?

This question extends RQ2 by examining whether the predictive value of within-person variability in response time is robust across student groups with varying levels of achievement. Prior research has suggested that the relationship between response time and performance may vary depending on students’ ability. To explore this, we grouped students based on their overall test scores and conducted separate profile analyses within each group. This allowed us to assess the stability of pattern and level effects across achievement levels. Importantly, our goal was not to estimate the total predictive effect of response time within subgroups or to perform moderation analysis by comparing regression coefficients across groups. Instead, we focused on whether the pattern effect (within-person variability) consistently contributed more to predictive power than the level effect (average response time) across all groups. In addition, to further illustrate that screen-level response times provide more nuanced and informative insights into students’ test-taking processes, we draw on theoretical perspectives from dual-process theory and test-taking theory, which explain how students may engage in different cognitive processes across various test screens.

## 3. Methods

### 3.1. Data and Sample

We obtained publicly available assessment data for the Problem Solving and Inquiry tasks (PSI tasks), which were administered as part of the Trends in International Mathematics and Science Study (TIMSS) 2019. The PSI tasks were time-constrained, computer-based tests designed to evaluate students’ higher-order thinking skills, particularly reasoning and applying skills in mathematics and science (Mullis et al. 2021). These tasks featured visually attractive, interactive scenarios with narratives or themes simulating real-world problems. As such, the PSI tasks represent an innovative item format that differs significantly from traditional assessment items. Student responses to the PSI tasks varied in format. In some cases, responses consisted of a single number, while in others, they included extended strings containing information about drawn lines or the dragging and dropping of objects. Additionally, the student response table contained typed responses, which were later transferred to the scoring system for human evaluation. This system also included screenshot images of responses generated using the line-drawing tool (Martin et al. 2020). Detailed information about the PSI tasks and their scoring procedures can be found on the TIMSS 2019 website: https://timss2019.org/psi/introduction/index.html (accessed on 15 April 2024).

This study focused on booklet ID 15 of the grade-four PSI mathematics assessment. In the assessment, 17 screens contained a total of 29 items distributed across 3 distinct scenarios (i.e., PSI tasks), while other screens introduced the items or provided clues. There were instances where a single screen presented multiple items. Therefore, screen response times were selected as a unit of analysis in this study because they represent the most granular and available variable linked to individual item response times. Screens that introduced the items or provided clues were excluded from the analyses because they were not involved in the problem-solving process.

The original sample consisted of 13,829 fourth-grade students from 30 countries, including 6724 girls and 6755 boys. Referencing the achievement benchmark adopted in TIMSS 2019 (Mullis et al. 2020), the students’ test performance was distributed as follows: 1453 students (10.51%) did not reach low achievement (i.e., very low), 2522 students (18.24%) reached low achievement, 4301 students (31.10%) reached intermediate achievement, 4088 students (29.56%) reached high achievement, and 1465 students (10.59%) reached advanced achievement. Students’ mathematics test performance was derived from a single continuous score based on their response accuracy on the PSI tasks, calculated using the scaling method described in the TIMSS 2019 Technical Report (Martin et al. 2020). The classification of students’ test performance did not aim for a precise interpretation of students’ achievement levels but rather to facilitate analyses to contribute to our understanding of how the relationship between response time and test performance may vary across different achievement levels.

### 3.2. Data Analysis

Descriptive statistics, including missing data analysis, were calculated first. Then, missing data were listwise deleted to facilitate the criterion-related profile analysis, which requires a complete matrix of observations across all screens. Listwise deletion was chosen over data imputation because the latter could introduce artificial patterns or assumptions about students’ test-taking behaviors or cognitive styles, potentially distorting the within-person variability central to our analysis. We report the percentage of missing data per screen and per student in the Results section to assess potential bias.

To facilitate comparisons of response times across screens, which contain items that inherently require different amounts of time to complete, we employed the z-score transformation. This involved subtracting the mean response time of all students on that screen from each student’s raw response time, then dividing the result by the standard deviation of students’ response time on that screen. The resulting z-scores represent standardized response times, with a mean of 0 and a standard deviation of 1. The standardized scores enable us to assess whether a student consistently maintained the same relative position, spending a similar amount of time on different items throughout the test compared to other students. In other words, each student would exhibit a consistent pattern of standardized scores across screens. The standardized response time data served as the unit of analysis for profile analyses.

To address RQ1, a one-sample profile analysis with Hotelling’s *T*^2^ was used to examine whether students’ average standardized response times across screens were statistically equivalent. Since response times were standardized, the analysis focused on relative timing patterns—that is, whether students, on average, responded faster or slower on certain screens relative to other screens. The null hypothesis of the analysis assumes that on average, students would spend equal standardized time across all screens, with no screen standing out as faster or slower relative to others. Following Bulut and Desjardins (2020), this hypothesis could be conceptualized as that the ratios of the observed means over their hypothesized means are all equal to 1, against the alternative hypothesis that at least one of the ratios is not equal to 1. In this context, the observed means represent the average standardized response time on each screen, while the hypothesized means represent the expected standardized response time under the assumption of no relative differences—that is, a value of zero for each screen, due to standardization. Mathematically, they could be expressed as

(1) $$H_{0}:\;\frac{\mu_{1}}{\mu_{1}^{0}}=\;\frac{\mu_{2}}{\mu_{2}^{0}}=\ldots =\;\frac{\mu_{p}}{\mu_{p}^{0}}=\;1\;H_{1}:\;\frac{\mu_{j}}{\mu_{j}^{0}}\neq 1,\;for\;j\in \;\{1,\;2,\;3,\;\ldots ,\;p\},$$

where *μ* is the observed means of the standardized response time for screens, *μ =* [*μ*_1_, *μ*_2_, *…*, *μ_p_*], whereas *μ*_0_ is the vector of hypothesized means for the 1st–*p*th screen, *μ*_0_ = [$\mu_{1}^{0}$, $\mu_{2}^{0}$, …, $\mu_{p}^{0}$]. In addition to testing whether the ratios are all equal to 1, profile analysis can also be used to directly test whether all ratios are equal. Thus, another pair of hypotheses could be proposed:

$$H_{0}:\;\frac{\mu_{1}}{\mu_{1}^{0}}=\;\frac{\mu_{2}}{\mu_{2}^{0}}=\ldots =\;\frac{\mu_{p}}{\mu_{p}^{0}}$$

(2) $$H_{1}:\;\mathrm{at}\;\mathrm{least}\;\mathrm{one}\;\mathrm{pair}\;\mathrm{of}\;\mathrm{ratios}\;\mathrm{is}\;\mathrm{not}\;\mathrm{equal}.$$

While repeated measures analysis of variance (ANOVA) could be an alternative analysis to test mean differences across screens in our study, it treats response time as a single construct measured repeatedly. In contrast, one-sample profile analysis conceptualizes screen-level response times as a multivariate pattern, without assuming that response times on different screens reflect the same underlying construct or process (Bulut and Desjardins 2020). This distinction is important for our study, as response time is not a stable trait but an outcome of student–task interactions with items that may vary across screens. Therefore, one-sample profile analysis offers a more flexible and conceptually appropriate framework for testing whether the pattern of response times is equivalent across screens.

To address RQs 2 and 3, this study employed criterion-related profile analysis, a regression-based statistical method developed by Davison and Davenport (2002), to quantify the predictive value of response time profiles. This method identifies profiles (i.e., combinations of subscores) correlated with a criterion variable. According to Davison and Davenport, when subscores are predictive, a specific criterion-related profile pattern emerges, such that it is associated with high scores on the criterion variable and minimizes prediction error. This criterion-related pattern can be described in terms of the linear regression coefficients and used to reveal the relationship between individual subscores and the criterion variable. Davison and Davenport have demonstrated that criterion-related profile analysis has the advantage of quantifying the predictive value of subscores beyond the total score. In this study, standardized screen response times were employed as the predictor subscores, and the criterion variable was students’ overall test performance. The following paragraphs outline Davison and Davenport’s procedures for identifying the criterion-related pattern and quantifying the predictive value of subscores.

In the first step, a multiple regression analysis is established to predict the criterion variable based on a composite of subscores. In general, multiple regression can be written as

(3) $$Y_{p}^{\prime }=\sum_{v}b_{v}X_{pv}+\;a,$$

where $Y_{p}^{\prime }$ represents the predicted criterion score for person *p*, $b_{v}$ denotes the regression coefficient for predictor subscore *v*, $X_{pv}$ represents person *p*’s subscore for predictor *v*, and *a* is the intercept constant. To determine the criterion-related pattern, the regression coefficients are subtracted by the mean regression coefficients identified in the multiple regression equation. Let the criterion-related pattern be b*, the criterion-related pattern vector can be mathematically expressed as $b^{*}=[b_{v}^{*}=b_{v}-\bar{b}]$, where $\bar{b}=\frac{1}{V}\sum_{v}b_{v}$.

The b* coefficients (also called the criterion-related pattern) must be interpreted in a configural manner; that is, each coefficient reflects a relative strength or weakness within the overall pattern (Davison et al. 2023). A screen with a positive b* indicates that, compared to other screens, spending more time on that screen contributes more to higher test performance. Conversely, a negative b* value suggests that spending less time on that screen leads to higher performance. Importantly, these coefficients are not interpreted in isolation; rather, they form a response time profile that is predictive of performance when a student’s timing pattern closely matches the criterion-related profile. In such cases, students whose response time profiles align more closely with the pattern tend to achieve higher test scores.

This interpretation differs from that of the original regression coefficients (*b*), which quantify the unique predictive contribution of each screen’s response time to test performance, controlling for time spent on other screens. In other words, while *b* values reflect between-student differences in how time on a specific screen predicts outcomes, b* values describe a contrast pattern that captures the within-student configuration of time allocation associated with high performance.

In addition to identifying the criterion-related pattern, the multiple regression analysis decomposes each person’s profile of predictor subscores into two components: a profile level effect and a profile pattern effect. The level effect is defined as the mean of the subscores for person *p*, while the pattern effect is defined as a vector consisting of the deviations between a person’s subscore and their mean score $X_{p.}$. Therefore, the level effect can be expressed as $X_{p.}=\frac{1}{V}\sum_{v}X_{pv}$, where *V* is the total number of subscores, and the pattern effect can be denoted as $X_{p}=[X_{pv}-X_{p.}]$. According to Davison and Davenport (2002), the pattern effect can be re-expressed as $Cov\left(b_{v},X_{pv}\right)=\;\sum_{v}\left(b_{v}-b.)(X_{pv}-X_{p.}\right)$.

The second step of criterion-related profile analysis involves estimating the variation of the criterion variable accounted for by the level and pattern effects. This is accomplished through another regression equation, which can be written as

(4) $$Y_{p}^{\prime }=b_{1}X_{p.}+b_{2}Cov(b_{p},\;X_{pv})+a,$$

where $X_{p.}$ is the mean of the predictor subscores for person *p* (i.e., level effect), ${Cov(b}_{p,}\;X_{pv})$ is the pattern effect, *b*_1_ is the regression coefficient of the level effect, and *b*_2_ is the regression coefficient of the pattern effect. Then, the regression equation allows us to examine whether pattern effects incrementally explain the variation in the criterion variable over level effects. This is analyzed through a series of hierarchical regressions, where the criterion variable is predicted by (1) the level effect alone, (2) the pattern effect alone, (3) the increment of the pattern effect above and beyond the level effect, and (4) the increment of the level effect above and beyond the pattern effect. *F* statistics and changes in *R*^2^ are used to evaluate improvements in model performance.

The third and last step of criterion-related profile analysis is to conduct cross-validation, which involves replicating the profile pattern and level effects in another sample. The purpose is to test the generalizability of the results. Following the procedure suggested by Davison and Davenport (2002), the full dataset is randomly split into two subsets of equal size. The criterion-related pattern (b*) obtained by analyzing one subset is then used to predict the criterion for the other subset, and vice versa. This cross-validation procedure aims to estimate the potential drop in explained variance (*R*^2^) when regression-derived criterion-related patterns are applied to a new sample drawn from the same population. It is important to note that this procedure differs from common cross-validation techniques used in machine learning (e.g., k-fold or holdout validation). In the context of profile analysis, the primary goal is not to optimize model parameters but rather to evaluate the replicability and stability of the predictor–criterion relationships across samples.

For all analyses described above, we chose not to apply a log transformation to the response time data, despite the presence of skewness in many screen-level response times. This decision was made primarily to preserve the interpretability of the response time profiles. Additionally, the one-sample profile analysis with Hotelling’s *T*^2^ test is essentially a multivariate extension of ANOVA, which is moderately robust to violations of multivariate normality, particularly in large samples (Sheng 2008; Wu 2009). This robustness reduces the likelihood of Type I error and supports the validity of our findings despite the skewed distributions. Furthermore, the criterion-related profile analysis, which relies on multiple regression techniques, assumes Gaussian errors but is also considered relatively robust to violations of multivariate normality (Knief and Forstmeier 2021). To assess the impact of using the original response time data (which were standardized but non-log-transformed), we conducted a sensitivity analysis using log-transformed response times. The results were highly consistent with those obtained from the original z-standardized data. Therefore, while we acknowledge the skewness in the response time distributions, we retained the original z-standardized values (based on non-log-transformed response times) for the main analysis and results presented in this manuscript.

This study employed the profileR package (Desjardins and Bulut 2020) in R (R Core Team 2023) to address the proposed RQs. Specifically, the paos function was used to test the two null hypotheses of one-sample profile analysis with Hotelling’s *T*^2^ (RQ1). Additionally, the cpa function with the default parameters (i.e., scalar constant = 100; Gaussian family with the “identity” model link function) was used to perform the criterion-related profile analysis for both the full sample (RQ2) and by each achievement group (RQ3). The function provided results for the estimated variation in test scores, which were explained by the level and pattern effects of response time.

## 4. Results

### 4.1. Descriptive Statistics

The descriptive statistics for the screen response time and test score for the full sample are presented in Table 1. The average screen response time ranges from 34.83 to 191.39 s, with standard deviations varying from 24.64 to 100.72 s. The interquartile range spanned from 22.39 to 121.52 s. Some screen response times show very large positive kurtosis values (e.g., Screen 7: 130.59 s; Screen 11: 31.33 s), distributed as having heavier tails and a more pronounced peak. This means that a large proportion of students spent a very long or short time on each screen. Correlation analysis revealed that the pairwise correlation coefficients of standardized response times across screens ranged from −0.22 to 0.41, indicating weak to moderate relationships between response times on different screens. Additionally, the correlation coefficients between individual standardized response times and test performance ranged from −0.25 to 0.44, suggesting varying degrees of association between response times and test scores.

**Figure 1:**
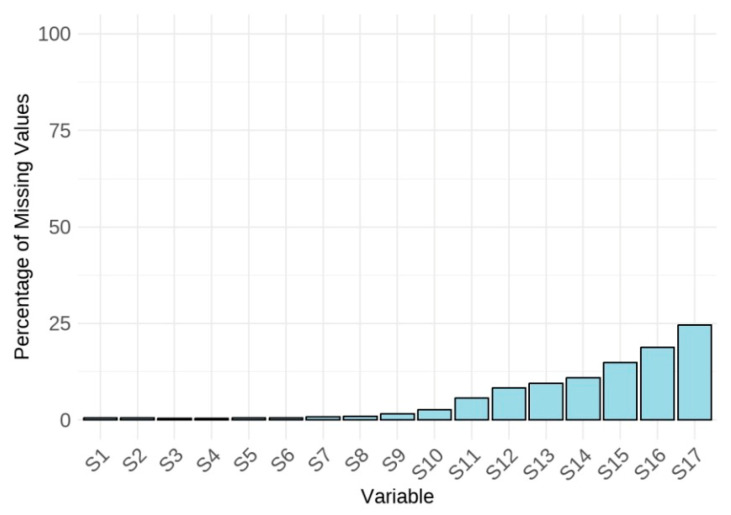
Missing percentage of response time for each screen.

**Figure 2:**
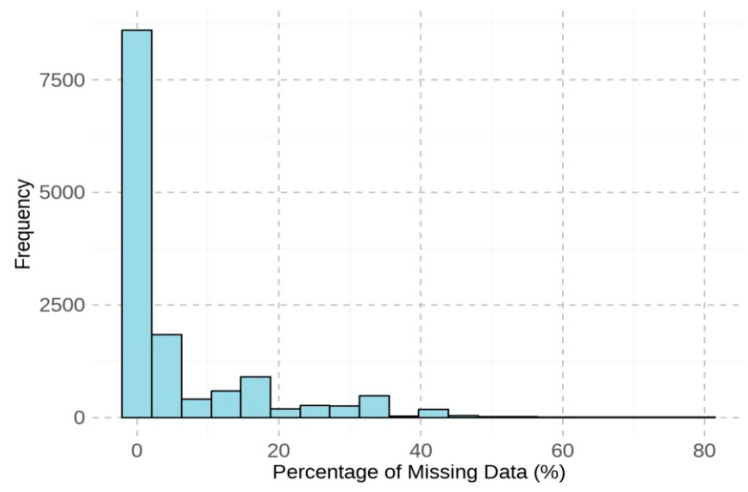
Histogram of missing data percentage for each student.

Figure 1 visualizes the percentage of missing values for each screen, revealing a trend of increasing missing data on screens toward the end of the test. Figure 2 presents a histogram of the percentage of missing values per student, showing that most students responded to all items, as indicated by a missing data percentage of 0. To facilitate the subsequent analyses that require complete data across all screens, students with any missing values were excluded. This listwise deletion resulted in a reduction of 25.33% of the total sample, leaving 10,326 students for the remaining analyses.

### 4.2. RQ1: Is There Meaningful Within-Person Variability in Students’ Response Times Across Test Screens?

According to the results of the one-sample profile analysis using Hotelling’s *T*^2^, both null hypotheses specified in the analysis were rejected. The first test rejected the null hypothesis that the ratio of the mean response time is 1 for each screen, *T*^2^ = 152,906.62, *F*(17, 10,309) = 8980.57, *p* < .001. The second test rejected the null hypothesis that the ratio of the mean response time is the same for each screen, *T*^2^ = 1452.63, *F*(16, 10,310) = 1452.63, *p* < .001. Therefore, it can be concluded that students’ response time did not remain constant across screens. As this study had no interest in determining which screen response time differs, post hoc comparisons were not performed.

### 4.3. RQ2: To What Extent Does Within-Person Variability in Response Time Predict Test Performance Beyond Average Response Time, and Does the Relationship Between Response Time and Test Performance Vary Across Screens?

RQ2 examines whether within-person variability in response time contributes to the prediction of test performance beyond what is explained by average response time. Specifically, we aim to quantify the predictive value of this variability and investigate whether individual screen response times differ in how they relate to test performance.

**Figure 3:**
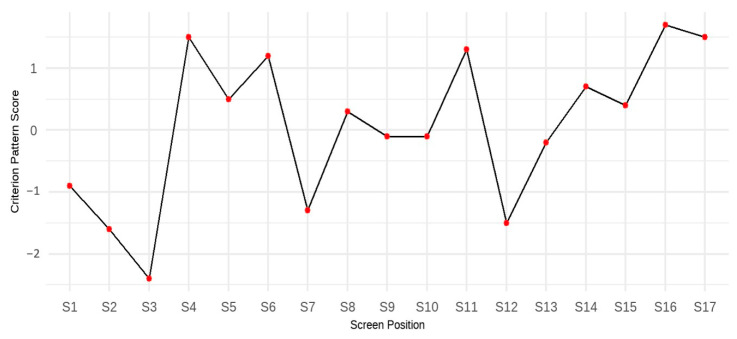
Criterion-related patterns for the full sample.

To begin, we analyzed the form and contribution of each screen-level response time. This step helps demonstrate that the relationship between response time and test performance is not constant across screens—for example, the assumption that faster students are consistently faster on every item does not hold. As part of the criterion-related profile analysis, a multiple regression equation was established using students’ test scores as the criterion variable and response times on 17 screens as predictors. Table 2 displays the regression coefficients (*b*) and the corresponding criterion-related patterns (b*). The results indicate that response times on individual screens are moderately strong predictors of test scores, with each response time uniquely contributing to the prediction. Figure 3 visualizes the criterion-related patterns across the 17 screens, showing 9 positive and 8 negative values. Rather than interpreting each value in isolation, the criterion-related pattern should be read as a whole: a positive b* value for a screen suggests that, within a student’s overall response time profile, spending more time on that screen tends to align with profiles associated with higher test scores. In contrast, a negative b* value indicates that spending less time on that screen, relative to time spent on others, is more typical of higher-performing students. Taken together, these coefficients describe the optimal configuration of time allocation across screens for achieving better performance. The variation in both *b* and b* values highlights that the predictive relationship between response time and performance varies across items or screens and in strength and direction. This further underscores the importance of analyzing within-person response time profiles rather than relying solely on aggregate measures such as average response time.

Next, we aim to quantify the predictive value of within-person variability in response time, which is a central focus of this study. In the criterion-related profile analysis, once the criterion-related pattern was identified, a subsequent multiple regression analysis decomposed the proportion of variation explained in the test score based on both level and pattern effects, as shown in Table 3. Specifically, the pattern effect alone accounted for 34.21% of the variance in test scores, while the level effect alone explained only 10.03% of the variance. Additionally, there was an incremental effect of the profile pattern effect and the level effect on each other, as the combined effect (i.e., full effect) explained significantly more variability in test score (*R*^2^ = 0.40; full effect vs. level effect: Δ*R*^2^ = 0.30, *p* < .001; full effect vs. pattern effect, Δ*R*^2^ = 0.06, *p* < .001). These results indicate that the predictive power of response time for test performance is largely attributed to the within-person variability, which refers to the change in standardized response time across items for the individual student.

Finally, we employed cross-validation to determine if the results obtained in the criterion-related profile analysis were generalizable. This procedure estimated the reduction in explained variance (*R*^2^) when criterion-related patterns derived from one sample were applied to another sample drawn from the same population, and vice versa. The results of the regression analysis examining the pattern, level, and full effects in the cross-validation are reported in Table 4. According to the findings, the criterion-related patterns and the proportion of variation in test scores explained were comparable between the two subsets, indicating a minimal drop in explained variance when applying the profile pattern to a new sample. These findings were consistent with those from the criterion-related analysis conducted for the full sample. Taken together, the results support the stability and replicability of the predictor–criterion relationships, suggesting that the findings of the criterion-related profile analysis were generalizable.

### 4.4. RQ3: Does Within-Person Variability in Response Time Consistently Predict Test Performance More Strongly than Average Response Time Across Different Achievement Levels? How Do Individual Screen-Level Response Times Relate to Performance Within Each Group?

To address RQ3, we examined whether the predictive value of within-person variability in response time holds consistently across different student achievement levels and whether response time profiles and criterion-related patterns differ between groups.

**Figure 4:**
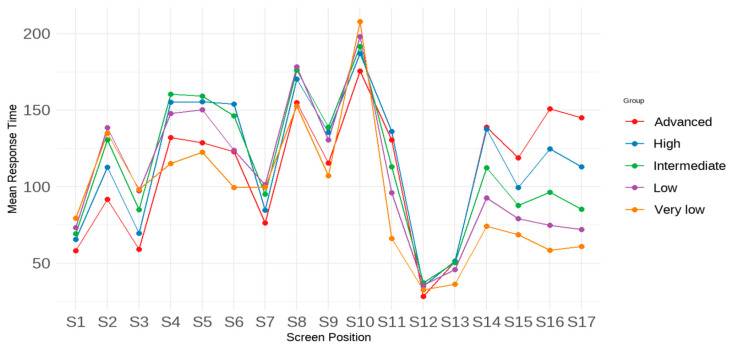
Profile means of response time (in seconds) across screens by achievement group.

First, the response time profile for each achievement group is visualized in Figure 4. Overall, a consistent trend emerges across all groups: students tend to spend more time on certain screens (e.g., Screens 8 and 10) and less time on others (e.g., Screens 12 and 13). However, the results also suggest that students with different achievement levels allocate their time differently. Specifically, students with lower test performance tended to spend more time on early screens and less time on later ones compared to their peers. Additionally, the time spent on Screens 4–6 by students with the lowest test performance deviated dramatically from that of other achievement groups. While all achievement groups spent the most time on Screen 10, the group with the lowest performance lingered the longest. These students dedicated significantly more time to later items compared to those with higher performances. Conversely, students with higher test performances spent less time on early items but more on later ones compared to groups with lower test performance. As for students with moderate achievement, their average response times were higher on Screens 4–9 and Screens 11–14 compared to students with either the highest or lowest performance.

We then examined how individual response times were associated with students’ test performance across different items, by achievement group. The regression coefficients of screen response times in predicting the test scores of students in each achievement group are presented in Table 5. A key insight from the results is that screen response times are associated with test performance in distinct ways for all groups. This underscores that relying on average response time alone would obscure important variability and lead to a substantial loss of predictive information.

**Figure 5:**
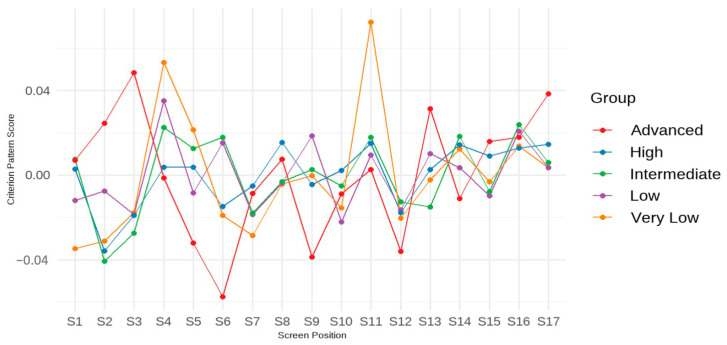
Criterion-related pattern by achievement group.

For example, according to this table, students’ achievement levels significantly impact the relationship between their response times on individual screens and their overall test performance, given that the regression coefficients (*b*) and criterion-related pattern (b*) are not always consistent across the five achievement groups. Specifically, for the students who neither reached low achievement nor attained high achievement, there were 11 positive regression coefficients and 6 negative regression coefficients. For the low and intermediate achievers, there are nine positive regression coefficients and eight negative regression coefficients. Lastly, the advanced achievement group saw only 6 positive regression coefficients and 11 negative regression coefficients. The associated criterion-related patterns for the achievement groups are also presented in Table 5 and visualized in Figure 5. It can be observed that in the advanced achievement group, investing more time in the first three screens was associated with higher scores, while spending more time on screens in the middle (e.g., Screens 6, 9, and 12) was linked to lower test scores. In contrast, for students in the lowest achievement group, spending more time on Screens 4 and 11 was associated with relatively higher scores. Consistent with the findings for RQ2, the variations in regression coefficients (*b*) and criterion-related patterns (b*) across screens and achievement groups suggests that using screen-level response time data provides richer insights for interpreting and explaining test performance than aggregated or average response time measures.

Finally, we tested the predictive value of within-person variability in response time within each achievement group. The results of the analysis examining level and pattern effects are presented in Table 6. Overall, the findings indicate that the proportion of variation in test score explained by the full effect for all achievement groups was significantly smaller than the full effect in the entire sample. The *R*^2^ values for the full effect ranged from 5% to 9%. The lower *R*^2^, compared to that in the full sample, is due to the fact that the classification of students into different achievement groups inherently accounted for considerable variation in test scores. Despite the lower overall *R*^2^, the pattern effects surpassed the level effects across all achievement groups. Notably, for all groups except those below the low benchmark (i.e., Very Low in Table 6), the variance explained by the level effect was close to zero. In contrast, the variance explained by the pattern effect was nearly equivalent to that of the full model. This suggests that the predictive value of response time for test score was mostly attributed to students’ within-person variability in response time rather than the average response time. Thus, the results demonstrate the advantage of using students’ individual screen response times to predict their test scores.

## 5. Discussion

### 5.1. RQ1: Is There Meaningful Within-Person Variability in Students’ Response Times Across Test Screens?

This study uses profile analysis to holistically examine students’ response time profiles and their relationship with the overall test performance. In response to the first RQ, the results suggest that students’ standardized response time varies across different screens. That is, in comparison to other students’ response times, an individual’s response time may fluctuate, such as taking more time on one item than others but taking less time on the next. This is an anticipated finding, given that response time is influenced by several personal factors, such as an individual’s ability (Wise 2017) and time management skills (Van Der Linden 2009). As motivation levels and test engagement vary across different test items (Wise and Kingsbury 2016), it is not surprising to observe fluctuations in an individual’s standardized response time. The results of the first RQ lay the groundwork for the remaining RQs, as they highlight the existence of within-person variability in response time.

### 5.2. RQ2: To What Extent Does Within-Person Variability in Response Time Predict Test Performance Beyond Average Response Time, and Does the Relationship Between Response Time and Test Performance Vary Across Screens?

Building on the first RQ, we further demonstrate that the predictive value of response time or test performance can be dissected into two effects using criterion-related profile analysis: the profile level effect and the pattern effect. Of these two, the pattern effect reveals the unique predictive value attributable to within-person variability in response time. Notably, the pattern effect exceeds the level effect. Therefore, this study substantiates that within-person variability explains a unique and larger portion of the variance in overall test performance, which is not accounted for by the average or total response time. The criterion-related pattern of response time in predicting overall test performance also highlights the value of using subscores to predict a criterion variable in the context of response time. This result provides evidence that the relationship between response time and performance varies across items throughout the test.

As indicated by the regression coefficients and criterion-related patterns, each response time predicts overall test performance differently in terms of strength and direction. These results are not surprising given that both item-specific factors and personal factors collectively influence response time, and such complex interplay can yield inconsistent relationships between response time and performance across different items. For instance, more capable students tend to answer difficult questions more quickly and effectively than their counterparts (Van Der Linden 2009). In this case, a shorter response time might correspond to better performance. Conversely, capable students often invest more time in later items in the test than other students, implying a negative correlation between response time and performance. Additionally, some items in the test may be challenging or tricky, requiring a sufficient amount of consideration and thinking before arriving at the correct answer. Therefore, dedicating more time to these items could be linked to higher performance. Due to the research objectives of this study, we did not aim to identify the specific reasons for the inconsistent relationship between response time and test performance. Importantly, our analyses provide a lens through which to examine how separate response times and the within-person variability in response time could offer more information to infer the cognitive processes and test-taking behaviors of students in the assessment.

### 5.3. RQ3: Does Within-Person Variability in Response Time Consistently Predict Test Performance More Strongly than Average Response Time Across Different Achievement Levels? How Do Individual Screen-Level Response Times Relate to Performance Within Each Group?

Our results indicate that the advantage of using response time profiles to predict overall test performance is consistent across different achievement groups, as the pattern effect always significantly exceeds the level effect. In addition, similar to the findings of RQ2, within-person variability provides valuable information for inferring students’ cognitive processes and test-taking behaviors during the assessment. To illustrate, within-person variability can be used to interpret the relationship between response time and overall test performance across different achievement groups. For instance, our results suggest that high-achieving students generally spend less time on initial screens and items but allocate more time to later ones. In contrast, low achievers tend to devote more time to early items and less to those later in the test. One possible explanation for this pattern, as proposed by the dual-process theory (Shiffrin and Schneider 1977), is that more capable students may possess automatic processes or the skills and knowledge needed to quickly identify the correct solution. Conversely, students who rely more on controlled processes may eventually deplete their cognitive resources, leading to reduced motivation and decreased ability to carefully evaluate later items (Sweller et al. 1998; Wise and Smith 2011). The reduction in response time may, therefore, be attributed to the extent of disengagement. Another reason could be that less capable students spend excessive time on early items or screens, resulting in time pressure to complete the later items.

Another result from the profile analyses is the distinct criterion-related patterns observed across achievement groups. These results provide further insight into how the relationship between response time and overall test performance varies across groups. For example, among advanced achievers, a shorter amount of time spent on items is more likely to be associated with better performance. This may be explained by the fact that advanced achievers tend to employ more automated processes, resulting in less time spent on items compared to other advanced achievers, reflecting their proficiency in the tested knowledge materials and abilities. Conversely, for students who achieved the lowest performance, investing more time in items generally correlates with improved performance. The reason behind this may be that students who achieved the lowest performance might have depleted their cognitive resources and experienced fatigue throughout the test event. Therefore, dedicating more time to items may indicate that students remain engaged in solution behavior rather than resorting to rapid guessing. These results highlight the value of using separate response time data to better explain students’ test performance.

### 5.4. Implications

Response time, a key type of process data, has become an invaluable source for understanding students’ cognitive processes and test-taking behaviors during assessment events. Research has highlighted the role of response time in revealing underlying cognitive strategies, effort levels, and engagement throughout testing (Goldhammer et al. 2014; Lee and Jia 2014). This study’s findings underscore the predictive value of using distinct response times for individual screens or items, as they provide granular insights into students’ behaviors, including within-person variability, which has been shown to increase the explained variance in overall test performance. The consistency of our results, supported by cross-validation and analyses across achievement groups, suggests that within-person variability in response time is a robust predictor of performance, irrespective of students’ proficiency levels. This pattern is likely to generalize to other assessment types and student populations, as response time fluctuations can similarly reflect shifts in cognitive strategies or effort allocation (Ratcliff et al. 2015; Van Der Linden 2009). However, the magnitude of these effects may vary across contexts. For instance, younger students (e.g., elementary graders) might exhibit more pronounced within-person variability due to developing self-regulation skills, whereas older adults could display distinct patterns tied to cognitive load management (Naumann and Goldhammer 2017). Students may demonstrate different effort allocation patterns in high-stakes assessment contexts, potentially influencing both the pattern and predictive value of within-person variability (Wise and Smith 2011). To fully establish generalizability, future studies may replicate our analyses using datasets from diverse assessments and populations.

Furthermore, disaggregated response times provide a foundation for examining how students engage with test items, drawing on theories such as test-taking behavior and dual-process theory. According to dual-process theory, individuals employ fast, intuitive responses and slower, more deliberate reasoning in decision-making (Evans and Stanovich 2013). This study’s use of separate response times aligns with this theoretical perspective, as it allows for a more nuanced view of test engagement—identifying instances where students may engage in intuitive or deliberate processing across different items. The data, therefore, supports more valid interpretations of test scores by revealing the diversity of cognitive strategies students employ—a factor that has been increasingly recognized as critical to understanding test validity (Kane 2013).

The findings of this study also have implications for the design and administration of educational assessment. Echoing the words of Maddox from OECD (Maddox 2023), we are shifting from regarding process data as a ‘by-product’ of digital assessment to intentionally incorporating it ‘by design.’ Embracing this perspective will enable educational assessments not only to uncover what students know but also to deepen our understanding of how they engage with the test-taking process and the factors affecting their performance. Hence, future learning and assessment systems should contemplate gathering more detailed data, such as item-level response times, to facilitate a more thorough comprehension of the interaction among response time, item characteristics, and student performance on a granular level. This kind of data is invaluable for research in learning analytics, paving the way for a more personalized learning and assessment experience for students. For instance, computerized adaptive tests (CATs) can adjust the difficulty level of subsequent questions based on students’ response time to maintain their test motivation and engagement (e.g., Bulut et al. 2023a; Choe et al. 2018; Gorgun and Bulut 2023). The findings of this study suggest that one should avoid assuming that longer response times always indicate students’ difficulties in solving a question. As the relationship between response time and test performance varies across different items and achievement groups, it is important to identify the reasons for longer or shorter response times to better inform the item selection algorithm for CATs.

### 5.5. Limitations and Future Research

This study has several limitations. The primary limitation stems from the extensive missing response time data in the TIMSS PSI dataset, particularly for later items and screens (Mullis et al. 2021). To conduct the criterion-related profile analysis, which requires complete data across all screens, we excluded students with any missing response time data through listwise deletion. While this approach preserves the authenticity of within-person variability by avoiding artificial imputation of behavioral patterns, it may also introduce selection bias and limit the generalizability of the findings. Specifically, students excluded due to missing data may differ from those retained. For example, the missing data could relate to students’ difficulties in meeting the assessment’s time constraints, as some students may have spent excessive time on earlier items, resulting in insufficient time to complete later questions (i.e., not reached items). Moreover, given that the TIMSS PSI tasks are considered low-stakes, students might not have engaged with the assessment fully, potentially leading to more missing responses (i.e., omitted items). This missing data may reflect disengagement due to fatigue or awareness that the assessment has limited personal consequences. Future research should explore the factors contributing to missing data and their effects on students’ response time profiles and criterion-related patterns across items. For instance, studies conducted in high-stakes contexts or assessments without time might reveal distinct response time profiles and criterion-related patterns from those observed here. Investigating these differences will help determine the extent to which findings on within-person variability in response time can be generalized across assessment contexts.

Another limitation is that while this study primarily focused on within-person variability in response times and its utility in predicting test performance, it did not examine the potential impact of item-level or test-level characteristics, such as item type, difficulty, cognitive processes involved, assessment domains, interactive elements embedded within the PSI tasks, and the sequencing of items by difficulty. A comprehensive analysis of these characteristics could provide further insights into the variability of students’ response times across items and differences relative to their peers. Future studies could produce additional insights by investigating these characteristics, thereby enhancing understanding of their impact on response time profiles and test performance.

## Figures and Tables

**Table 1:** Descriptive statistics of screen response times and test scores (full sample).

Variable	*M*	*SD*	*IQR*	Skewness	Kurtosis
S1	68.79	51.32	43.07	3.50	21.65
S2	123.12	74.53	67.80	3.23	21.23
S3	81.30	55.27	46.30	3.58	24.92
S4	148.90	98.20	100.43	2.34	11.32
S5	149.38	97.96	86.65	2.58	11.91
S6	137.13	100.70	96.82	2.48	10.84
S7	91.66	57.77	49.63	6.85	130.59
S8	170.21	100.72	114.15	1.56	4.76
S9	130.57	78.06	80.81	1.88	6.88
S10	191.39	97.52	107.37	1.43	4.40
S11	113.98	72.94	77.62	2.89	31.33
S12	34.83	24.64	22.39	3.95	37.48
S13	48.47	31.18	29.80	2.81	19.20
S14	115.44	70.93	74.22	1.83	7.44
S15	91.35	52.65	54.68	1.93	8.72
S16	103.27	68.27	86.46	1.54	9.83
S17	95.40	69.03	80.63	1.61	5.01
Test Score	520.17	89.91	121.52	−0.36	−0.06

Note. The unit of response time is in seconds.

**Table 2:** Regression coefficients and the associated criterion-related patterns (full sample).

Screen	*b*	*S.E.*	*t*	b*
S1	−0.04	0.01	−3.50	−0.09
S2	−0.10	0.01	−9.06	−0.16
S3	−0.19	0.01	−16.79	−0.24
S4	0.21	0.01	17.50	0.15
S5	0.10	0.01	8.82	0.05
S6	0.18	0.01	14.78	0.12
S7	−0.07	0.01	−6.46	−0.13
S8	0.09	0.01	8.71	0.03
S9	0.04	0.01	4.09	−0.01
S10	−0.05	0.01	−5.34	−0.01
S11	0.19	0.01	19.08	0.13
S12	−0.09	0.01	−10.78	−0.15
S13	0.03	0.01	3.96	−0.02
S14	0.12	0.01	12.95	0.07
S15	0.09	0.01	10.61	0.04
S16	0.23	0.01	24.05	0.17
S17	0.21	0.01	22.75	0.15

Note: *b* = regression coefficient, b* = criterion-related pattern. All *t* values are statistically significant at *α* = 0.001.

**Table 3:** Hypothesis testing for the changes of *R*^2^ (full sample).

Hypotheses	Δ*R*^2^	df_1_	df_2_	*F*
*R*^2^_full_ = 0	0.40	17	10,308	41.58
*R*^2^_pattern_ = 0	0.34	16	10,308	334.94
*R*^2^_level_ = 0	0.10	1	10,308	1150.07
*R*^2^_full_ = *R*^2^_level_	0.30	16	10,308	319.19
*R*^2^_full_ = *R*^2^_pattern_	0.06	1	10,308	965.77

Note: *R*^2^_full_: the proportion of variance in the criterion variable explained by the full model. *R*^2^_pattern_: the proportion of variance explained by the pattern effect. *R*^2^_level_: the proportion of variance explained by the level effect. All *F* values are statistically significant at *α* = 0.001.

**Table 4:** Hypothesis testing for the changes of *R*^2^ in cross-validation.

Hypotheses	Δ*R*^2^	df_1_	df_2_	*F*
*R*^2^_full_ = 0				
Random Sample 1	0.41	1	5161	3542.16
Random Sample 2	0.39	1	5159	3230.03
*R*^2^_pattern_ = 0				
Random Sample 1	0.34	1	5162	2677.83
Random Sample 2	0.34	1	5160	2624.81
*R*^2^_level_ = 0				
Random Sample 1	0.11	1	5162	643.44
Random Sample 2	0.09	1	5160	510.83
*R*^2^_full_ = *R*^2^_level_				
Random Sample 1	0.30	1	5162	2578.05
Random Sample 2	0.30	1	5160	2474.82
*R*^2^_full_ = *R*^2^_pattern_				
Random Sample 1	0.09	1	5162	569.55
Random Sample 2	0.09	1	5160	401.57

Note: *R*^2^_full_: the proportion of variance in the criterion variable explained by the full model. *R*^2^_pattern_: the proportion of variance explained by the pattern effect. *R*^2^_level_: the proportion of variance explained by the level effect. All *F* values are statistically significant at *α* = 0.001.

**Table 5:** Regression coefficient and the criterion-related patterns by achievement groups.

Screen	Very Low		Low		Intermediate		High		Advanced	
	*b_v_*	b*	*b_v_*	b*	*b_v_*	b*	*b_v_*	b*	*b_v_*	b*
S1	−0.02	−0.03	−0.01	−0.01	0.01	0.01	0.01	0.00	0.00	0.01
S2	−0.01	−0.03	0.00	−0.01	−0.04	−0.04	−0.03	−0.04	0.01	0.02
S3	0.00	−0.02	−0.01	−0.02	−0.02	−0.03	−0.01	−0.02	0.04	0.05
S4	0.07	0.05	0.04	0.04	0.03	0.02	0.01	0.00	−0.01	0.00
S5	0.04	0.02	0.00	−0.01	0.02	0.01	0.01	0.00	−0.04	−0.03
S6	0.00	−0.02	0.02	0.02	0.02	0.02	−0.01	−0.01	−0.07	−0.06
S7	−0.01	−0.03	−0.01	−0.02	−0.01	−0.02	0.00	−0.01	−0.02	−0.01
S8	0.01	0.00	0.00	0.00	0.00	0.00	0.02	0.02	0.00	0.01
S9	0.02	0.00	0.02	0.02	0.01	0.00	0.00	0.00	−0.05	−0.04
S10	0.00	−0.02	−0.02	−0.02	0.00	−0.01	0.01	0.00	−0.02	−0.01
S11	0.09	0.07	0.01	0.01	0.02	0.02	0.02	0.02	−0.01	0.00
S12	0.00	−0.02	−0.01	−0.02	−0.01	−0.01	−0.01	−0.02	−0.05	−0.04
S13	0.02	0.00	0.01	0.01	−0.01	−0.02	0.01	0.00	0.02	0.03
S14	0.03	0.01	0.01	0.00	0.02	0.02	0.02	0.01	−0.02	−0.01
S15	0.01	0.00	−0.01	−0.01	0.00	−0.01	0.01	0.01	0.00	0.02
S16	0.03	0.01	0.03	0.02	0.03	0.02	0.02	0.01	0.01	0.02
S17	0.02	0.00	0.01	0.00	0.01	0.01	0.02	0.01	0.03	0.04

Note: *b* = regression coefficient, b* = criterion-related pattern.

**Table 6:** Explained variance (*R*^2^) in test scores by full, pattern, and level effects for each achievement group.

Effect	Very Low	Low	Intermediate	High	Advanced
*R* ^2^_full_	0.09	0.06	0.06	0.05	0.09
*R* ^2^_pattern_	0.04	0.05	0.06	0.05	0.07
*R* ^2^_level_	0.04	0.00	0.00	0.00	0.01
*R*^2^_full_–*R*^2^_level_	0.05	0.06	0.06	0.05	0.08
*R*^2^_full_–*R*^2^_pattern_	0.05	0.01	0.00	0.00	0.02

Note: Cell value represents *R*^2^. *R*^2^_full_: the proportion of variance in the criterion variable explained by the full model. *R*^2^_pattern_: the proportion of variance explained by the pattern effect. *R*^2^_level_: the proportion of variance explained by the level effect. *R*^2^_full_–*R*^2^_level_: additional variance explained by the pattern effect beyond the level effect. *R*^2^_full_–*R*^2^_pattern_: additional variance explained by the level effect beyond the pattern effect.

## Data Availability

This study used a publicly available dataset obtained from the TIMSS 2019 website: https://timss2019.org/international-database/index.html.

## Abbreviations

The following abbreviations are used in this manuscript:

ANOVA	Analysis of variance
PSI	Problem solving and inquiry
TIMSS	Trends in International Mathematics and Science Study
ISAR	Intraindividual speed–ability-relation
CAT	Computerized adaptive test
